# Acceptance of diagnosis and management satisfaction of patients with “suspected Lyme borreliosis” after 12 months in a multidisciplinary reference center: a prospective cohort study

**DOI:** 10.1186/s12879-023-08352-3

**Published:** 2023-06-06

**Authors:** Alice Raffetin, Amal Chahour, Julien Schemoul, Giulia Paoletti, Zhuoruo He, Elisabeth Baux, Solène Patrat-Delon, Steve Nguala, Pauline Caraux-Paz, Costanza Puppo, Pauline Arias, Yoann Madec, Sébastien Gallien, Julie Rivière

**Affiliations:** 1 Department of Infectious Diseases, Tick-Borne Diseases Reference Center of Paris and the Northern Region, General Hospital of Villeneuve-Saint-Georges, Villeneuve-Saint-Georges, France; 2 EpiMAI Research Unity, Laboratory of Animal Health, Ecole Nationale Vétérinaire d’Alfort, Anses-National Veterinaty School of Alfort, Maison-Alfort, France; 3 DYNAMIC Research Unity, UPEC-Anses, Créteil, France; 4 Department of Rheumatology, Tick-Borne Diseases Reference Center of Paris and the Northern Region, General Hospital of Villeneuve-Saint-Georges, Villeneuve-Saint-Georges, France; 5 Department of Psychiatry, Tick-Borne Diseases Reference Center of Paris and the Northern Region, General Hospital of Villeneuve-Saint-Georges, Villeneuve-Saint-Georges, France; 6 Department of Public Health, University of Paris Saclay, Saclay, France; 7 Department of Infectious Diseases, Tick-Borne Diseases Reference Center of the Eastern Region, Brabois Hospital, University Hospital of Nancy, Nancy, France; 8 Department of Infectious Diseases, Tick-Borne Diseases Reference Center of the Western Region, University Hospital of Rennes, Rennes, France; 9 Department of Psychology, Lumière University Lyon II, UMR 1296, Lyon, France; 10 Epidemiology of Emerging Diseases Unit, Institut Pasteur, University of Paris, Paris, France; 11 Department of Infectious Diseases, UH Henri Mondor, Créteil, France

**Keywords:** Lyme borreliosis, Multidisciplinary management, Satisfaction, Concordance, Diagnostic acceptance

## Abstract

**Introduction:**

Because patients with a “suspicion of Lyme borreliosis (LB)” may experience medical wandering and difficult care paths, often due to misinformation, multidisciplinary care centers were started all over Europe a few years ago. The aim of our study was to prospectively identify the factors associated with the acceptance of diagnosis and management satisfaction of patients, and to assess the concordance of the medical health assessment between physicians and patients 12 months after their management at our multidisciplinary center.

**Methods:**

We included all adults who were admitted to the Tick-Borne Diseases Reference Center of Paris and the Northern Region (TBD-RC) (2017–2020). A telephone satisfaction survey was conducted 12 months after their first consultation. It consisted of 5 domains and 13 items rated between 0 (lowest) and 10 (highest grade): (1)Reception; (2)Care and quality of management; (3)Information/explanations given to the patients; (4)Current medical condition and acceptance of the final diagnosis; (5)Overall appreciation. Factors associated with diagnosis acceptance and management satisfaction at 12 months were identified using logistic regression models. The concordance of the health status as assessed by doctors and patients was calculated using a Cohen’s kappa test.

**Results:**

Of the 569 patients who consulted, 349 (61.3%) answered the questionnaire. Overall appreciation had a median rating of 9 [8;10] and 280/349 (80.2%) accepted their diagnoses. Patients who were “very satisfied” with their care paths at TBD-RC (OR = 4.64;CI95%[1.52–14.16]) had higher odds of diagnosis acceptance. Well-delivered information was strongly associated with better satisfaction with the management (OR = 23.39;CI95%[3.52–155.54]). The concordance between patients and physicians to assess their health status 12 months after their management at TBD-RC was almost perfect in the groups of those with confirmed and possible LB (κ = 0.99), and moderate in the group with other diagnoses (κ = 0.43).

**Conclusion:**

Patients seemed to approve of this multidisciplinary care organization for suspected LB. It helped them to accept their final diagnoses and enabled a high level of satisfaction with the information given by the doctors, confirming the importance of shared medical decisions, which may help to reduce health misinformation. This type of structure may be useful for any disease with a complex and controversial diagnosis.

**Supplementary Information:**

The online version contains supplementary material available at 10.1186/s12879-023-08352-3.

## Key messages

What is already known on this topic?

- Among patients with a suspicion of Lyme borreliosis (LB), less than 15% have confirmed LB, and more than 80% a differential diagnosis, confirming the need for multidisciplinary structures.
- To our knowledge, there are not any studies about the satisfaction of the patients with these proposed multidisciplinary care paths.

What this study adds?

- Acceptance of diagnosis (80.2%) was associated with satisfaction with the care paths and the current medical condition of the patients.
- The high satisfaction with the information given by the doctors was a key element of the management satisfaction, confirming the importance of shared medical decisions to meet the patients’ expectancies and reduce misinformation.

How this study might affect research, practice and policy?

- Provided that this satisfaction survey is externally assessed by additional studies, it could be useful to regularly assess patients’ satisfaction in the context of multidisciplinary management for suspected LB, and these multidisciplinary structures might be generalized for other complex diseases.

**Supplementary Information:**

The online version contains supplementary material available at 10.1186/s12879-023-08352-3.

## Introduction

Lyme borreliosis (LB) is the most common tick-borne disease in Europe and in the USA. It is caused by spirochetes of the *Borrelia burgdorferi* sensu lato complex [1, 2]. Diagnosis of LB associates an exposure to tick bite, the presence of specific defined-LB manifestations (the most frequent being erythema migrans (EM) and Lyme neuroborreliosis) and a positive microbiological test (serological and sometimes PCR tests, save for EM); none of them alone makes the diagnosis of the infection certain [3–5]. European guidelines recommend a mono-antibiotic therapy for LB treatment. The therapy should be given for 14 to 28 days according to the infection’s stage and its clinical manifestation [6, 7]. No studies have yet proven the clinical benefit of a longer antibiotic treatment [8–12].

The diagnosis and the management of LB may be challenging for several reasons: (i) its wide range of clinical pictures, sometimes resembling other pathologies; (ii) the rare sequelae that may occur mainly after late disseminated LB, with most of patients being completely cured within one month to three years in the most complicated cases [13–19]; and (iii) the possible presence of subjective symptoms (asthenia, polyalgia, cognitive complaints) at all stages of the disease [14, 20], which may persist after a well-adapted treatment, producing the post-treatment Lyme disease syndrome (PTLDS) [13, 14, 20, 21], with no clear guidelines for their management. The causative role of LB in these subjective symptoms is a source of questions insofar as these non-specific symptoms may be encountered in the course of other infectious (Epstein-Barr-Virus, SARS-CoV-2, etc.) or non-infectious diseases [22]). In addition, some patients are referred for antibiotic therapy for a suspicion of LB, sometimes at their own request, but are finally diagnosed with other diseases, mainly rheumatological, neurological, auto-immune or psychological [23–27].

Therefore, many patients suspected of having LB may experience diagnosis wandering and difficult care paths, often due to misinformation. To improve the health care organization of LB, a French national care plan for LB was started in 2016 that favored the creation of multidisciplinary LB centers. These centers are joint endeavors between departments of infectious diseases, internal medicine, rheumatology, neurology, algology, dermatology, psychiatry, microbiology, and physical rehabilitation to manage patients presenting a suspicion of LB, in a multidisciplinary approach. There, challenging cases are discussed in monthly multidisciplinary consultation meetings. One such clinic opened in December 2017 at the General Hospital of Villeneuve-Saint-Georges in suburban Paris, France. This center was labeled the Tick-borne Diseases Reference Center (TBD-RC) for Paris and the Northern region in July 2019 by the French Ministry of Health, which also established four other such clinics in France. Teams in other countries have also initiated such care organizations since 2010 [23–25], showing a European awareness of the need for the management of complex LB and its differential diagnoses. These multidisciplinary experiences have revealed a low prevalence of confirmed LB (between 10 and 20%), and the multiplicity of the differential diagnoses [23–27]. We have previously demonstrated that the majority of patients (80.7%), independently of their final diagnoses, had favorable clinical outcomes one year after their first consultation at TBD-RC of Paris and the Northern region. However, the opinions of the patients about these multidisciplinary structures, their diagnosis acceptance, especially in spite of another diagnosis than LB, and their own health status assessment after receiving care in this type of multidisciplinary structure have not been studied yet [27]*.*

The aims of our study were to analyze the satisfaction levels of patients experiencing a multidisciplinary management for suspected LB at TBD-RC of Paris and the Northern region, to identify the factors associated with their diagnosis acceptance and their global satisfaction with the management, and to assess the concordance of the medical health assessment between the physicians and the patients 12 months after their first consultation at TBD-RC.

## Methods

We conducted a prospective descriptive and analytic cohort study, including all adults who consulted at TBD-RC of Paris and Northern Region for a suspicion of LB, from 1 December 2017 to 1 December 2020. We followed the STROBE guidelines [28] (Additional file 1).

### Population, setting, and intervention

**Fig. 1:**
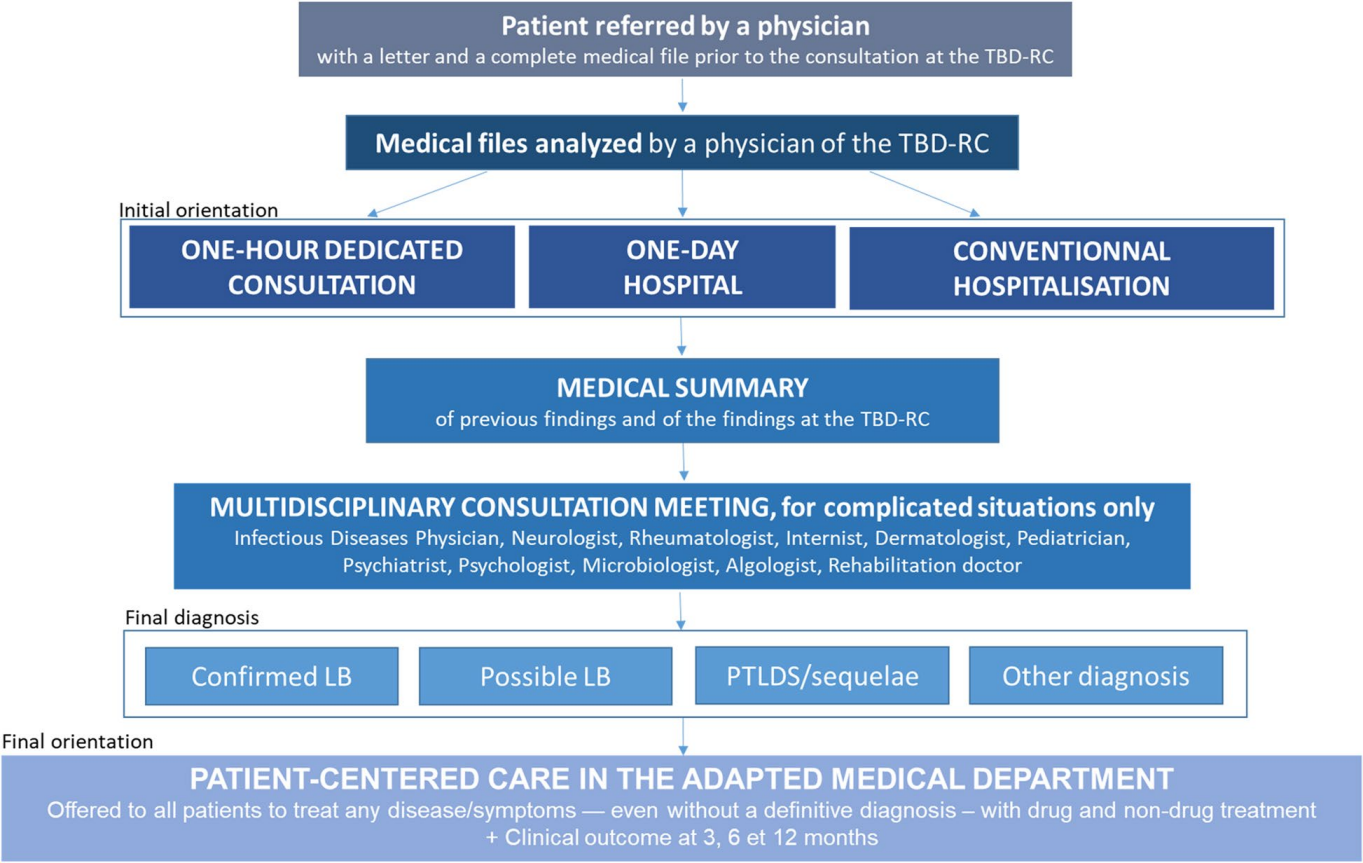
Care path of the patients consulting for a suspicion of LB at TBD-RC. TBD-RC = Tick-Borne Disease Reference Center; LB = Lyme borreliosis; PTLDS = Post-Treatment Lyme Disease Syndrome

The care path at TBD-RC was previously described [27] and is summarized in Fig. 1.

Patients with diagnoses associated with LB were classified as [13, 29]: confirmed LB (tick exposure, typical clinical signs and a positive serological test); possible LB (tick exposure and/or prior erythema migrans, evocative clinical signs and marked clinical improvement after 21 days of antibiotics); and post-treatment Lyme disease syndrome (PTLDS) (asthenia/polyalgia/cognitive complaints) or sequelae (objective impairment) after a confirmed LB treated as recommended. PTLDS and sequelae were pooled together as they are both responsible for persistent symptoms after treatment. Therefore, combining them together was clinically relevant. Moreover, as sequelae are very rare, the effective would have been too small to perform statistical tests separately. The other patients were classified in the group “other diagnoses,” which were made by a doctor specialized in the field. A final orientation in the adapted medical department was offered to every patient, independently of their final diagnosis.

A telephone-based satisfaction survey was conducted, independently from the staff consulting at TBD-RC, and pseudonymized, 12 months after the first consultation at TBD-RC.

To assess the current health condition of the patients 12 months after their management at TBD-RC, the physician in charge of the patient had a rating scale between 1 and 5. In the satisfaction survey, patients had a scale between 0 and 10. The current medical condition corresponded to: complete recovery (score 1 for physicians; score 9–10 in the satisfaction survey for patients), partial improvement consisting of persistent clinical signs or symptoms allowing resumption of daily and professional activities (score 2 for physicians; score 7–8 for patients), stagnation (score 3 for physicians; score 5–6 for patients), or deterioration (score 4 for physicians; score 0–4 for patients).

### Patient data and satisfaction survey

Patients’ data were routinely collected in standardized medical files at the TBD-RC, independently of the study, to ensure the correct follow-up of the patients.

The satisfaction survey comprised 15 items: 12 items rated between 0 (lowest grade) and 10 (highest grade), 1 item about the acceptance with 3 categories (yes, no, and partially), and 2 free-text items. These items covered five domains: (1) reception; (2) care and quality of management (by the medical team, by the paramedical team, responsiveness and compassion to patients, care path at TBD-RC); (3) information and explanations given to the patients; (4) current medical condition after the management at the TBD-RC compared to the previous one and acceptance of the final diagnosis; and (5) overall appreciation (Additional file 2). This questionnaire was inspired by the MedRisk instrument, and adapted to our setting (multidisciplinary management for the suspicion of LB) [30, 31]. It was presented and discussed with patients’ associations involved in LB on the one hand, and in other diseases such as HIV or diabetes on the other hand, to check whether this survey was adequate to their expectations. Their suggestions were taken into account to improve the questionnaire.

### Statistical analysis

The four groups of patients classified according to their final diagnosis as assessed at the TBD-RC of Paris and the Northern region (i.e. confirmed LB, possible LB, PTLDS or sequelae, and other diagnosis) were previously compared according to socio-demographic, clinical, and microbiological characteristics, and 12-month outcomes after multidisciplinary care [27]. In the present study, we compared the satisfaction levels in the four groups of patients at 12 months after the first consultation at TBD-RC. Moreover, we focused on the group “other diagnoses” to analyze more precisely the results in patients with a bodily distress syndrome and in patients without a specific diagnosis, as the diagnostic wandering could remain.

Categorical variables are reported here as proportions and percentages, and continuous variables as medians with interquartile ranges (IQR). Categorical variables were compared by chi-squared or Fischer’s exact test, as appropriate. Continuous variables were compared between groups by ANOVA or Kruskal–Wallis test, as appropriate.

Factors associated with the acceptance of the final diagnosis (yes vs partially or no) and those with satisfaction with the management (yes for a score ≥ 7 and no for a score < 7) were identified using logistic regression models. In both analyses, factors associated with the outcome with a *p*-value < 0.25 in univariate analysis were considered in the multivariate model. For the acceptance of the diagnosis, we chose “care and quality of management by the medical team” to avoid collinearity with the other variables and thus make them irrelevant to the multivariate model. For the satisfaction with the management, we focused on the medical management only, which seemed more relevant, especially as we then studied the concordance of the health status assessed by doctors and patients. A stepwise backward procedure was then applied to identify factors that remained independently associated with the outcome. Gender and age were forced in the models.

The concordance was calculated using a simple Cohen’s kappa test (deterioration/stagnation versus partial improvement/recovery). A sensitivity analysis was performed with a weighted Cohen’s kappa (deterioration, stagnation, partial improvement, and recovery). The strength of agreement was defined as “slight” for a Cohen’s kappa between 0 and 0.20, “fair” for one between 0.21 and 0.40, “moderate” for one between 0.41 and 0.60, “substantial” for one between 0.61 and 0.80, and “almost perfect” for one between 0.81 and 1.00.

A *p-*value < 0.05 was defined for statistical significance. All analyses were performed using Stata version 16 (College Station, Texas, USA).

The analyses of the two free-text items will be performed in another study using qualitative methods.

### Approval of the ethics committee

The local ethics committee of the University Hospital of Créteil, France, approved this research (N°2021–02-03). All the included patients (or their legal guardian(s)) gave an informed consent to the use of their medical data for research purposes, prior to their management at TBD-RC of Paris and the Northern region and to the satisfaction questionnaire. The research sponsor signed a commitment to comply with the “Reference Methodology MR004” of the French Data Protection Authority (CNIL, 2,216,096 v 0, December 10, 2019).

### Funding

None.

## Results

**Fig. 2:**
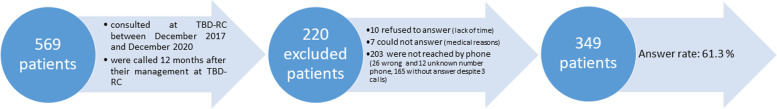
Flow chart of the patients who were solicited to answer the satisfaction questionnaire at 12 months

Of 569 patients admitted to the TBD-RC of Paris and the Northern region between December 2017 and December 2020, 349 (61.3%) answered the satisfaction questionnaire (Fig. 2).

### Characteristics of the patients

No statistical difference was found between the characteristics of patients who answered and those who did not answer the satisfaction questionnaire (Additional file 3). Characteristics of those who answered the satisfaction questionnaire are presented in the Table 1. The median age was 48 years old, and 71.4% of the patients were practicing forest-based leisure activities. There were statistically more patients with a history of tick-bite (*p* = 0.001) or EM (*p* < 0.001) in the three groups with a diagnosis associated with LB. The duration of the symptoms before the initial consultation at TBD-RC was statistically longer in patients with another diagnosis (*p* < 0.001). Of note, 10.3% of the patients self-referred to the center with a complete medical file but with no letter from a physician. They were admitted as they were in medical wandering. Most of the patients (66.5%) had symptoms for more than six months, except in the group of confirmed LB patients, who had a significantly shorter duration of symptoms (*p* < 0.001). Only 31.8% of the patients had a positive serology in ELISA and Western-Blot, regardless of the final diagnosis. Most of the patients (65.3%) had received at least one antibiotic therapy before the first consultation at TBD-RC and 17.5% had received a non-recommended one (exceeding eight weeks or associating different molecules).

**Table 1:** Comparison of the characteristics of the four groups of patients who answered the satisfaction questionnaire

**Characteristics of the patients**	**Total** ***N* = 349 (%)**	**Confirmed LB** ***N* = 48 (%)**	**Possible LB** ***N* = 31 (%)**	**PTLDS or sequelae** ***N* = 34 (%)**	**Other diagnoses** ***N* = 236 (%)**	***P* value**
**Age, (years), median [IQR]**	48 [35,62]	48 [35,62]	49 [35,62]	48 [35,62]	48 [35,61]	0.242
**Male**	146 (41.8)	30 (62.5)	16 (51.6)	9 (26.5)	91 (38.6)	0.003
**Lifestyle**						0.287
Home in a rural area	72 (20.6)	6 (12.5)	10 (32.3)	7 (20.6)	49 (20.8)	-
Employment in rural areas/forest	17 (4.9)	2 (4.2)	1 (3.2)	0 (0.0)	14 (5.9)	-
Forest-based leisure activities	249 (71.4)	40 (83.3)	20 (64.5)	26 (76.5)	163 (69.1)	-
No exposure	11 (3.2)	0 (0.0)	0 (0.0)	1 (2.9)	10 (4.2)	-
**Past history of tick-bite**	234 (67.1)	40 (83.3)	25 (80.7)	28 (82.4)	141 (59.8)	0.001
**Past history of erythema migrans**	97 (27.9)	29 (60.4)	11 (35.5)	16 (48.5)	41 (17.4)	< 0.001
**Patients referred by a physician** **with a letter**	313 (89.7)	46 (95.8)	30 (96.8)	30 (88.2)	207 (87.7)	0.108
General Practitioner	241 (69.1)	31 (64.6)	26 (83.9)	26 (76.5)	158 (67.0)	
Specialist physician	59 (16.9)	11 (22.9)	2 (6.5)	4 (11.8)	42 (17.8)	
Emergency unit physician	13 (3.7)	4 (8.3)	2 (6.5)	0 (0.0)	7 (3.0)	
No letter, patient self-referral	36 (10.3)	2 (4.2)	1 (3.2)	4 (11.8)	29 (12.3)	
**Duration (days) of chief complaints prior to consultation at TBD-RC, median [IQ 25,75]**	425.5 [140.5, 1208.5]	406.5 [135, 1171]	422 [139, 1191]	425.5 [144, 1208.5]	532.5 [167.5, 1456.5]	< 0.001
**Patient's chief complaint**						< 0.001
Erythema migrans	10 (2.9)	6 (12.5)	0 (0.0)	1 (2.9)	3 (1.3)	
Clinical signs/symptoms evoking early disseminated LB (< 6 months)	100 (28.7)	27 (56.3)	12 (38.7)	10 (29.4)	51 (21.6)	
Clinical signs/symptoms evoking late disseminated LB (> 6 months)	232 (66.5)	15 (31.3)	19 (61.3)	23 (67.7)	175 (74.2)	
Questions after a tick-bite	4 (1.2)	0 (0.0)	0 (0.0)	0 (0.0)	4 (1.7)	
Positive serological test with no clinical signs	3 (0.9)	0 (0.0)	0 (0.0)	0 (0.0)	3 (1.3)	
**Serological test**						< 0.001
IgM and/or IgG positive in ELISA and WB	111 (31.8)	33 (68.8)	12 (38.7)	19 (55.9)	47 (19.9)	
IgG positive in ELISA only	46 (13.2)	5 (10.4)	8 (25.8)	5 (14.7)	28 (11.9)	
IgM and IgG negative in ELISA	163 (46.7)	7 (14.6)	11 (35.5)	10 (29.4)	135 (57.2)	
No serology (suspicion of erythema migrans)	26 (7.5)	3 (6.3)	0 (0.0)	0 (0.0)	23 (9.8)	
**Antibiotic therapy prescribed before TBD-RC**	228 (65.3)	36 (75.0)	16 (51.6)	34 (100.0)	142 (60.2)	< 0.001
Antibiotic therapy > 4 weeks	71 (20.3)	12 (25.0)	2 (6.5)	14 (41.2)	43 (18.2)	0.003
Non-recommended treatments (> 8 weeks of antibiotics and/or associated antimicrobials)	61 (17.5)	6 (12.5)	0 (0.0)	10 (29.4)	45 (19.1)	0.011

*LB* Lyme borreliosis, *PTLDS* Post-Treatment Lyme Disease Syndrome, *IQR* Inter quartile range, *ELISA* Enzyme-Linked Immunosorbent Assay, *WB* Western-Blot, *TBD-RC* Tick-Borne Diseases Reference Center

### Descriptive analyses of the satisfaction survey

**Fig. 3:**
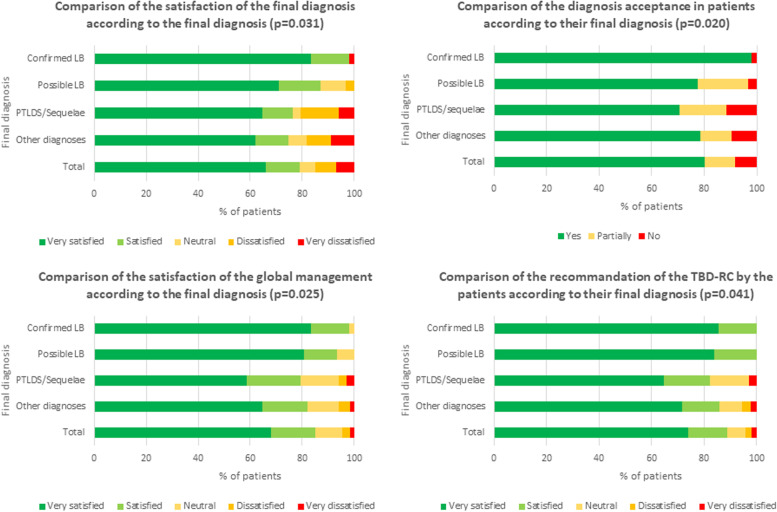
Comparative results of the overall appreciation between the 4 groups of patients at 12 months

The answer rate was not different between the four groups of patients (*p* = 0.44). The overall median (IQR) appreciation score was 9 [8;10] (Table 2). Overall, 276 (79.1%) patients were satisfied with their final diagnosis (score ≥ 7), 280 (80.2%) accepted their final diagnoses, 296 (84.8%) were satisfied with the management and 310 (88.8%) recommended the TBD-RC (Fig. 3). Scores were significantly higher among patients with a confirmed LB than among patients with other diagnoses, except when it came to the assessment of their health condition. Those scores did not differ from those of the other groups of patients (*p* = 0.18).

**Table 2:** Comparative results of the satisfaction questionnaire between the 4 groups of patients at 12 months

Domains and Items rated from 0 (worst) to 10 (best) Median, [25;75]	Total *N* = 349 (%)	Confirmed LB *N* = 48 (%)	Possible LB *N* = 31 (%)	PTLDS or Sequelae *N* = 34 (%)	Other Diagnoses *N* = 236 (%)	*P*-Value
**Domain 1: Reception**						
Satisfaction of the reception by the secretary	9 [8;10]	9 [8;10]	9 [8;9]	8 [8;9]	8 [8;10]	0.017
**Domain 2: Care and quality of management**						
By the paramedical team	9 [8;10]	9 [9;10]	9 [8;9]	8 [7;9]	9 [8;10]	0.007
By the medical team	9 [8;10]	10 [9;10]	9 [9;10]	9 [8;10]	9 [8;10]	0.011
Responsiveness and compassion to patients	9 [8;10]	10 [9;10]	9 [9;10]	9 [8;10]	9 [8;10]	0.023
Care-path at TBD-RC	9 [8;10]	9 [8;10]	9 [8;10]	8 [7;10]	9 [8;10]	0.020
**Domain 3: Information and explanations given to the patients**						
By the secretary	9 [8;10]	9 [8;10]	9 [8;10]	8 [7;10]	8 [8;10]	0.003
By the paramedical team	9 [8;10]	9 [9;10]	9 [8;9]	8 [7;9]	9 [8;10]	0.004
By the medical team	9 [8;10]	10 [9;10]	9 [9;10]	9 [8;10]	9 [8;10]	< 0.001
**Domain 4: Current medical condition**						
** Current condition after the management at the TBD-RC compared to the previous one**	8 [7;9]	8.5 [8;9]	8 [7;9]	8 [7;9]	8 [7;9]	0.185
Acceptance of the final diagnosis						0.020
* Yes*	280/349 (80.2)	47/48 (97.9)	24/31 (77.4)	24/34 (70.6)	185/236 (78.4)	
* No*	29/349 (8.3)	1/48 (2.1)	1/31 (3.2)	4/34 (11.8)	23/236 (9.8)	
* Partially*	40/349 (11.5)	0/48 (0.0)	6/31 (19.4)	6/34 (17.7)	28/236 (11.9)	
**Domain 5: Overall appreciation**						
Satisfaction of the final diagnosis	9 [8;10]	10 [9;10]	9 [8;10]	9 [8;10]	9 [6;10]	0.031
Satisfaction of global management	9 [8;10]	10 [9;10]	9 [9;10]	9 [8;10]	9 [8;10]	0.025
Recommendation of the TBD-RC to your surroundings	9 [8;10]	10 [9;10]	9 [9;10]	9 [8;10]	9 [8;10]	0.041

*LB* Lyme borreliosis, *PTLDS* Post-Treatment Lyme Disease Syndrome, *TBD-RC* Tick-Borne Diseases Reference Center

The scores evaluating reception, the care, and the quality of the management provided by the paramedical team one the one hand and by the medical team on the other hand, the responsiveness and the compassion to the patients, the care path at TBD-RC, and the information given by the doctor were significantly higher among patients with a confirmed LB than among patients with other diagnoses (*p* = 0.008, *p* = 0.009, *p* = 0.001, *p* = 0.004, *p* = 0.005, *p* < 0.001, respectively).

Patients with a confirmed LB had significantly better evaluations of their care paths at TBD-RC than patients with PTLDS/sequelae (*p* = 0.010). Patients with confirmed LB accepted their diagnosis significantly better than patients with a possible LB (*p* = 0.006), PTLDS/sequelae (*p* = 0.001), or other diagnoses (*p* = 0.006). Satisfaction with the final diagnosis and with the global management were significantly better in patients with confirmed LB compared to other diagnoses (both *p* = 0.004). Patients with confirmed LB recommended the TBD-RC significantly more than patients with other diagnoses (*p* = 0.009).

Moreover, patients oriented in the care paths of infectious diseases, rheumatology, neurology, internal medicine, or general practice had a significant better acceptance of the diagnosis than patients oriented in psychology or psychiatry (*p* < 0.001), and had a higher level of satisfaction with the management at TBD-RC than patients oriented in psychology or psychiatry (*p* = 0.009).

Among the “other diagnoses” group, we focused on patients with a bodily distress syndrome who answered the satisfaction questionnaire (*n* = 30): 17 (56.7%) accepted their diagnosis, 6 (20.0%) partially accepted their diagnosis, and 7 (23.3%) refused the diagnosis; 15 (50.0%) were very satisfied with the management, 4 (13.3%) were satisfied, 9 (30.0%) were moderately satisfied and 2 (6.7%) were not satisfied; 18 (60.0%) strongly recommended the TBD-RC, 6 (20.0%) recommended TBD-RC, 4 (13.3%) had no opinion, and 2 (6.7%) did not recommend TBD-RC.

Finally, we also focused on patients with no specific diagnosis at the end of the investigations at the TBD-RC (*n* = 17): 16 (94.1%) accepted their diagnosis and 1 (5.9%) did not; 15 (88.2%) were very satisfied with the management at TBD-RC, 1 (5.9%) was satisfied and 1 (5.9%) was moderately satisfied; 15 (88.2%) strongly recommended the TBD-RC, 1 (5.9%) recommended TBD-RC and 1 (5.9%) did not.

### Factors associated with the diagnostic acceptance and the management satisfaction at 12 months

In the multivariate analysis (Additional file 4), patients “very satisfied” with their care paths at TBD-RC had higher odds (OR = 4.64, 95% confidence interval (CI) [1.52–14.16]) of diagnosis acceptance compared to patients only “satisfied.” Patients with a possible LB had lower odds of diagnosis acceptance compared to patients with other diagnoses (OR = 0.23, 95%CI [0.07–0.77]). Patients “moderately satisfied” with the care and the management of the doctors at TBD-RC had lower odds of diagnosis acceptance compared to satisfied patients (OR = 0.05, 95%CI [0.01–0.32]). Patients assessing their current medical state compared to the previous one as “stagnation” had lower odds of diagnosis acceptance compared to patients describing a “partial improvement” (OR = 0.16, 95%CI [0.06–0.42]).

In the multivariate analysis about management satisfaction (Additional file 5), patients over 48 years had marginally significant higher odds of satisfaction with management (OR = 31.98, 95%CI [1.79–571.74], *p* = 0.051) than patients under 35. Patients “very satisfied” with the information given by the doctors had higher odds of satisfaction with management than “satisfied” patients (OR = 23.39, 95%CI [3.52–155.54]). Patients who were moderately satisfied with their care and management by the medical team had lower odds of satisfaction with management (OR = 0.01, 95%CI [0.00–0.10]) such as patients moderately satisfied with the care paths (OR = 0.01, 95%CI [0.00–0.08]), compared to satisfied patients. Gender, final diagnosis, responsiveness, and compassion to patients were not associated with satisfaction with management. Notably, in the univariate analysis, a first line of antibiotics prescribed at the TBD-RC was significantly associated with a better satisfaction with management compared to that of patients who had received a previous one (OR = 2.59, 95%CI [1.17–5.71], *p* = 0.011). However, a second line of antibiotics prescribed at TBD-RC was not associated with a better satisfaction (*p* = 0.124).

### Concordance of the medical health assessment between the physicians and the patients 12 months after the management at TBD-RC

**Fig. 4:**
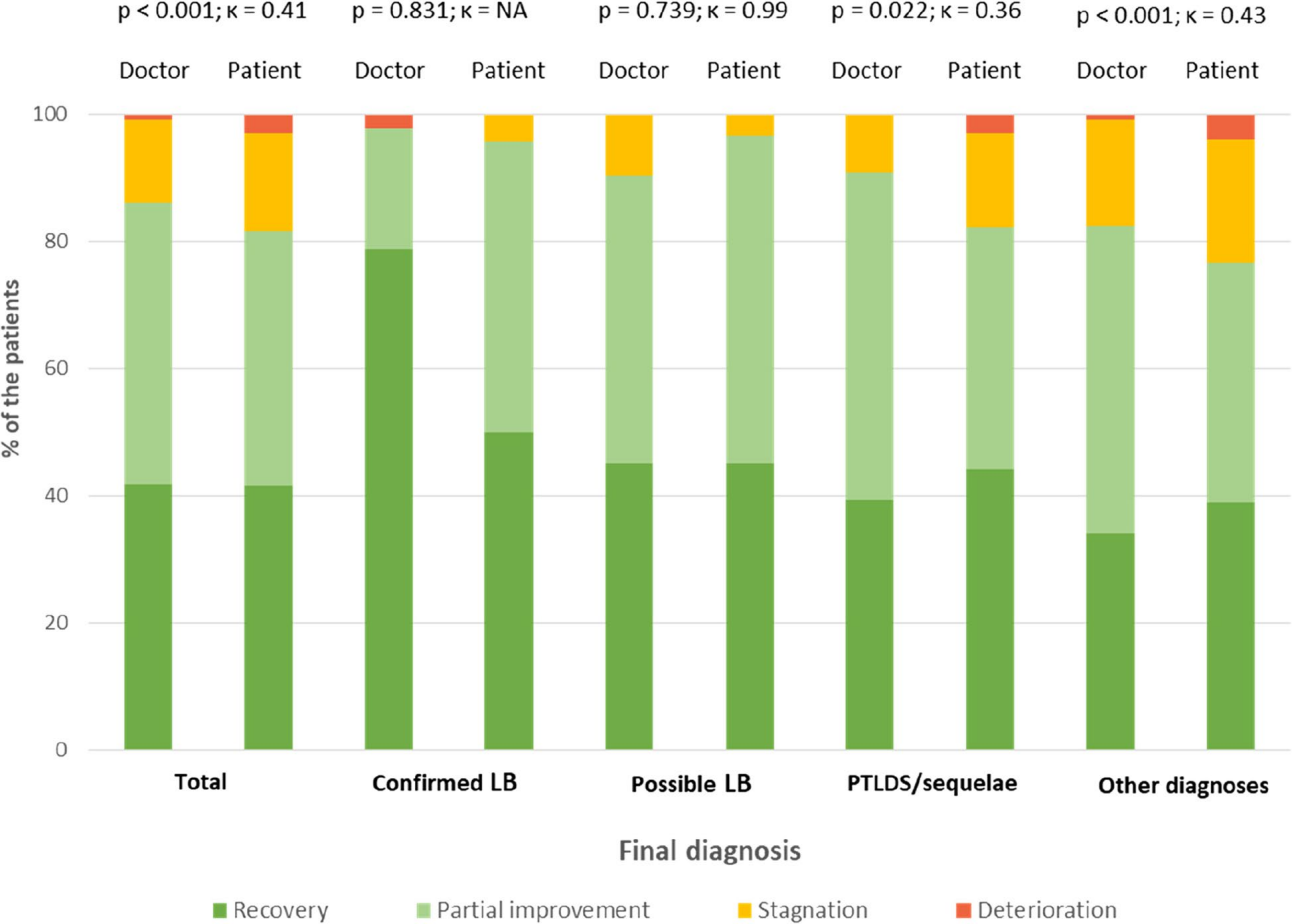
Comparison of the medical health assessment between the physicians and the patients at 12 months

The Cohen’s kappa value in all the patients demonstrated a moderate agreement (κ = 0.41) between the doctor and the patient health assessment at 12 months after their management at TBD-RC (Table 3 and Fig. 4). Nonetheless, there was no difference between the doctor and the patient health assessment in patients with confirmed LB or possible LB, with a Cohen’s kappa value showing an almost perfect agreement in patients with possible LB (κ = 0.99). In patients with PTLDS/sequelae, the agreement was fair (κ = 0.36), and in patients with other diagnoses it was moderate (κ = 0.43). The differences in agreement were always in the same direction: patients assessed their health status more severely than physicians did (Table 3). There was no difference in the results of the simple Cohen’s kappa and of the weighted Cohen’s kappa.

**Table 3:** Concordance of the medical health assessment between the physicians and the patients at 12 months

Current medical condition of the patients at M12 after TBD-RC, compared to the previous medical condition	Assessed by the patient	Assessed by the physician	*P*-value	Cohen’s Kappa
**Of all the patients**			< 0.001	0.41
Deterioration (score 0–4) Or Stagnation (score 5–6)	63/345 (18.3)	48/345 (13.9)		
Partial improvement (score 7–8) Or Recovery (score 9–10)	282/345 (81.7)	297/345 (86.1)		
**Of patients with a confirmed LB**			0.831	NA
Deterioration (score 0–4) Or Stagnation (score 5–6)	2/47 (4.3)	1/47 (2.1)		
Partial improvement (score 7–8) Or Recovery (score 9–10)	45/47 (95.7)	46/47 (97.9)		
**Of patients with a possible LB**			0.739	0.99
Deterioration (score 0–4) Or Stagnation (score 5–6)	1/31 (3.2)	3/31 (9.7)		
Partial improvement (score 7–8) Or Recovery (score 9–10)	30/31 (96.8)	28/31 (90.3)		
**Of patients with PTLDS/sequelae**			0.022	0.36
Deterioration (score 0–4) Or Stagnation (score 5–6)	6/33 (18.2)	3/33 (9.1)		
Partial improvement (score 7–8) Or Recovery (score 9–10)	27/33 (81.8)	30/33 (90.9)		
**Of patients with other diagnoses**			< 0.001	0.43
Deterioration (score 0–4) Or Stagnation (score 5–6)	54/234 (23.1)	41/234 (17.5)		
Partial improvement (score 7–8) Or Recovery (score 9–10)	180/234 (76.9)	193/234 (82.5)		

*NA* Not adapted, Cohen’s kappa could not be performed because of too close values but there was no statistical differences (almost perfect accordance), *LB* Lyme borreliosis, *PTLDS* Post-Treatment Lyme Disease Syndrome

Moreover, despite moderate agreement in patients with other diagnoses regarding their orientation in the adapted department, there was not any statistical difference between the medical and the patients’ assessments of the patients’ health statuses (*p* = 0.083).

## Discussion

### Summary of the principal findings

To our knowledge, this is the first study assessing the diagnostic acceptance and the satisfaction of patients undergoing multidisciplinary management of suspected LB. We recorded a very good overall appreciation (median of 9/10 [8;10]) from the patients who consulted at the TBD-RC of Paris and the Northern Region. Overall, 79.1% (*n* = 276/349) of them were satisfied with the final diagnosis, 80.2% (*n* = 280/349) accepted their diagnosis, 84.8% (*n* = 296/349) were satisfied with the management and 88.8% (*n* = 310/349) recommended the TBD-RC to others. As expected, patients with confirmed LB showed significantly higher satisfaction level than patients with other diagnoses. Patients with a high satisfaction with the care paths at TBD-RC were four times more likely to accept their diagnosis. The high satisfaction of the information given by the doctors was the main factor positively associated with satisfaction with the management. The concordance between patients and physicians to assess their health status 12 months after their first consultation at TBD-RC was almost perfect in patients with confirmed and possible LB, fair in those with PTLDS/sequelae and moderate in those with other diagnoses.

### Strengths and weaknesses of the study

Our data should help other physicians involved in LB management and its differential diagnoses to better understand the expectations of patients and to improve their care paths. The comparison of the current medical condition assessed by patients or by physicians at 12 months after their management at TBD-RC also highlights the differences between “disease” and “illness” and might enable a better assessment of the latter in the future, leading to a better patient-centered care. Moreover, we obtained a high answer rate (61.3%), enhancing the power of the analyses, probably due to the three systematic reminders, and to the brevity of the questionnaire (2–5 min), which was highlighted by patients as a condition to answer.

The main limitation is that we used a non-previously validated satisfaction questionnaire for the management of LB in a monocenter study. Nonetheless, we assume that this point represents also a strength, as ours is the first study assessing this topic with a questionnaire drawn up by a dedicated and multidisciplinary team, by patients, and by patients’ associations to fulfill their expectations. We assume an innovative use of this survey. A multicenter validation of this questionnaire in other TBD-RC in France and in Europe could enable researchers to assess its external validity and its reproducibility in other settings.

### Strengths and weaknesses in relation to other studies

To our knowledge, the satisfaction of patients with a suspicion of LB had not been assessed before this study. Some studies have already been published and found similar results for other diseases (cancer, diabetes, etc.) or for specific settings (private care structures, rural hospitals, etc.) [32–35]. A study exploring the satisfaction of patients with health care in Chinese public hospitals in urban and rural areas demonstrated that the most important factors were the professional competence, communication/information, caring attitude, emotional support, and the environment/facilities [32]. Moreover, for the elderly care in private structures, satisfaction with care has come to play a crucial role. Kazemi et al*.* identified that supportive leadership was positively associated with satisfaction with care, as it enabled the job satisfaction of the workers and therefore a higher quality of the care delivered to the patients [33]. In addition, Moreno et al. showed that the satisfaction of patients with cancer care was associated with a high perception of their quality of life, and with a good communication with their care provider, as in our study [34]. In the future, we could implement our satisfaction survey with a question about the quality of life of the patients.

### Meaning of the study and implication for practice and for policy makers

#### Diagnostic certainty as an element of diagnostic acceptance and concordance? Not only and not necessarily

Patients with a possible LB had lower odds of accepting their final diagnosis compared to other diagnoses. The word “possible” introduces the notion of uncertainty, leading to a possible doubt about the final diagnosis. Actually, due to uncertainty, auto-diagnosis could be elaborated and shaped by patients’ emotions, representations and experiences of the disease. Consequently, “disease” (doctor’s point of view), “sickness” (societal point of view) and “illness” (patient’s point of view) can coexist, according to the different points of view [36]. Nonetheless, this point is balanced by the high satisfaction with the management at TBD-RC reported by patients with a possible LB, and by the results among patients with no specific diagnosis who reported a high acceptance of the absence of specific diagnosis and a high satisfaction with the global management at TBD-RC.

#### A favorable clinical outcome as element of agreement between doctors and patients

Despite the lower odds of diagnostic acceptance in patients with possible LB, patients with confirmed and possible LB had an almost perfect strength of concordance with the doctor’s health state evaluation after one year of management at TBD-RC. The better clinical outcomes of these two groups of patients (91.6% and 90.7% of patients with a favorable outcome, respectively) [27] seems to draw these two evaluations closer, by bringing together the “disease,” the “sickness” and the “illness” independently of the degree of subjectivity of the patients. A favorable outcome should also restore confidence in the health care system and free oneself from misconceptions.

#### Information, a key for the management satisfaction

The subjectivity of patients and their experiences of the disease have an important place in diagnosis acceptance, which follows the five well-known steps: the initial shock, the denial, the rebellion, the negotiation, the reflection and finally the acceptance [37]. Although information was well delivered, it was not associated with diagnosis acceptance by patients, probably because of their own experiences and their own “grief circles.” Other sources of information, such as media or the surroundings, can play a role in the construction of disease perception and representation. However, well-delivered information was strongly associated with better satisfaction with management, showing that the doctor-patient relationship is at the forefront of the care experience, and emphasizing the importance of the shared medical decision, as already demonstrated in other studies [32, 38]. Indeed, the time spent with the patient to share information and listen to them to meet their expectations may help to reduce medical wandering and health misinformation.

#### Multidisciplinary management to improve the satisfaction with global management

The high overall satisfaction with case management by a multidisciplinary team has been shown in our study, such as in other studies. Implementation of a pain management strategy in a trauma center in Australia involving a dedicated and multidisciplinary team led to improvements in communication about pain with the trauma patients and increased the patients’ pain satisfaction score [39]. This corroborates our results showing that the information was strongly associated with a better satisfaction, probably due to the fact that the TBD-RC had a dedicated and very specialized team. Moreover, in a multidisciplinary colorectal and uro-gynecology service in Ireland, seeing many specialists at the same place was associated with a high satisfaction of the patients and higher physician confidence [40].

### Unanswered questions and future research

More studies in other settings are warranted to assess these preliminary findings and the external validity of the satisfaction questionnaire used for LB. Studies in the field of social sciences and anthropology would be complementary, improving comprehension of the expectations of the patients, of their possible ensuing paradoxes, and of their points of satisfaction and dissatisfaction. They would also help to better understand the origins of misinformation that may have led to medical wandering and then to dissatisfaction of the TBC-RC. The type of satisfaction questionnaire we used in our center could be implemented after these warranted studies.

## Conclusion

Patients seemed to approve of this new multidisciplinary care organization for suspected LB, as in TBD-RC, showing high satisfaction with the diagnostic and therapeutic management. The final diagnostic acceptance was associated with the satisfaction with the proposed care paths and the current medical condition of the patients. The high satisfaction with the information given by the doctors was a key element of the satisfaction with the management, confirming the importance of the doctor-patient relationship and of the shared medical decision (time spent with patient to share information and to listen to them to meet their expectancies). This may help to reduce health misinformation.

The agreement between patients and physicians to assess their health status 12 months after their management at TBD-RC was almost perfect for patients with confirmed and possible LB, suggesting that a favorable clinical outcome allows for bringing these two evaluations closer, independently of the degree of subjectivity of the patients and of their degree of misconceptions.

Multidisciplinary structures may be useful for any complex diagnosis, such as LB, to help to reduce medical wandering and the negative impact of health misinformation.

## Supplementary Information

**Additional file 1: Supplementary file 1.** STROBE Statement—Checklist of items that should be included in reports of *cohort studies*.

**Additional file 2.** Satisfaction survey.

**Additional file 3: Supplementary file 2. **Comparison of the epidemiological characteristics of the patients who answered or not to the satisfaction survey, consulting the TBD-RC of Paris and the Northern region.

**Additional file 4. **Multivariate analyses of the associated factors with the diagnostic acceptance versus no acceptance at 12 months.

**Additional file 5. **Multivariate analyses of the associated factors with the management satisfaction versus no satisfaction at 12 months.

## Data Availability

The datasets used and/or analyzed during the current study are available from the corresponding author on reasonable request.

## References

[1] Schwartz AM, Hinckley AF, Mead PS, Hook SA, Kugeler KJ (2017). Surveillance for Lyme disease - United States, 2008–2015. MMWR Surveill Summ.

[2] Sykes RA, Makiello P (2017). An estimate of Lyme borreliosis incidence in Western Europe†. J Public Health Oxf Engl.

[3] Lohr B, Fingerle V, Norris DE, Hunfeld KP (2018). Laboratory diagnosis of Lyme borreliosis: current state of the art and future perspectives. Crit Rev Clin Lab Sci.

[4] Leeflang MMG, Ang CW, Berkhout J, Bijlmer HA, Van Bortel W, Brandenburg AH, et al. The diagnostic accuracy of serological tests for Lyme borreliosis in Europe: a systematic review and meta-analysis. BMC Infect Dis. 2016;16(1). Available from: http://bmcinfectdis.biomedcentral.com/articles/10.1186/s12879-016-1468-4. 10.1186/s12879-016-1468-4 PMC4807538 27013465

[5] Talagrand-Reboul E, Raffetin A, Zachary P, Jaulhac B, Eldin C (2020). Immunoserological diagnosis of human borrelioses: current knowledge and perspectives. Front Cell Infect Microbiol.

[6] Jaulhac B, Saunier A, Caumes E, Bouiller K, Gehanno JF, Rabaud C (2019). Lyme borreliosis and other tick-borne diseases. Guidelines from the French scientific societies (II). Biological diagnosis, treatment, persistent symptoms after documented or suspected Lyme borreliosis. Med Mal Infect.

[7] Nguala S, Baux E, Patrat-Delon S, Saunier F, Schemoul J, Tattevin P (2021). Methodological quality assessment with the AGREE II scale and a comparison of European and American guidelines for the treatment of Lyme borreliosis: a systematic review. Pathogens.

[8] Berende A, ter Hofstede HJM, Vos FJ, van Middendorp H, Vogelaar ML, Tromp M (2016). Randomized trial of longer-term therapy for symptoms attributed to Lyme disease. N Engl J Med.

[9] Berende A, Ter Hofstede HJM, Vos FJ, Vogelaar ML, van Middendorp H, Evers AWM (2019). Effect of prolonged antibiotic treatment on cognition in patients with Lyme borreliosis. Neurology.

[10] Fallon BA, Keilp JG, Corbera KM, Petkova E, Britton CB, Dwyer E (2008). A randomized, placebo-controlled trial of repeated IV antibiotic therapy for Lyme encephalopathy. Neurology.

[11] Klempner MS, Hu LT, Evans J, Schmid CH, Johnson GM, Trevino RP (2001). Two controlled trials of antibiotic treatment in patients with persistent symptoms and a history of Lyme disease. N Engl J Med.

[12] Krupp LB, Hyman LG, Grimson R, Coyle PK, Melville P, Ahnn S (2003). Study and treatment of post Lyme disease (STOP-LD): a randomized double masked clinical trial. Neurology.

[13] Stanek G, Fingerle V, Hunfeld KP, Jaulhac B, Kaiser R, Krause A (2011). Lyme borreliosis: clinical case definitions for diagnosis and management in Europe. Clin Microbiol Infect.

[14] Rupprecht TA, Birnbaum T, Pfister HW (2008). Pain and neuroborreliosis: significance, diagnosis and treatment. Schmerz Berl Ger.

[15] Müllegger RR, Glatz M (2008). Skin manifestations of lyme borreliosis: diagnosis and management. Am J Clin Dermatol.

[16] Eikeland R, Mygland Å, Herlofson K, Ljøstad U (2013). Risk factors for a non-favorable outcome after treated European neuroborreliosis. Acta Neurol Scand.

[17] Eikeland R, Mygland A, Herlofson K, Ljøstad U (2011). European neuroborreliosis: quality of life 30 months after treatment. Acta Neurol Scand.

[18] Stupica D, Lusa L, Ruzić-Sabljić E, Cerar T, Strle F (2012). Treatment of erythema migrans with doxycycline for 10 days versus 15 days. Clin Infect Dis.

[19] Strle K, Stupica D, Drouin EE, Steere AC, Strle F (2014). Elevated levels of IL-23 in a subset of patients with post-Lyme disease symptoms following erythema migrans. Clin Infect Dis.

[20] Zimering JH, Williams MR, Eiras ME, Fallon BA, Logigian EL, Dworkin RH (2014). Acute and chronic pain associated with Lyme borreliosis: clinical characteristics and pathophysiologic mechanisms. Pain.

[21] Nemeth J, Bernasconi E, Heininger U, Abbas M, Nadal D, Strahm C (2016). Update of the Swiss guidelines on post-treatment Lyme disease syndrome. Swiss Med Wkly.

[22] Brodin P, Casari G, Townsend L, O’Farrelly C, Tancevski I, Löffler-Ragg J (2022). Studying severe long COVID to understand post-infectious disorders beyond COVID-19. Nat Med.

[23] Coumou J, Herkes EA, Brouwer MC, van de Beek D, Tas SW, Casteelen G (2015). Ticking the right boxes: classification of patients suspected of Lyme borreliosis at an academic referral center in the Netherlands. Clin Microbiol Infect.

[24] Jacquet C, Goehringer F, Baux E, Conrad JA, Ganne Devonec MO, Schmutz JL (2019). Multidisciplinary management of patients presenting with Lyme disease suspicion. Med Mal Infect.

[25] Gynthersen RMM, Tetens MM, Ørbæk M, Haahr R, Fana V, Hansen K (2021). Classification of patients referred under suspicion of tick-borne diseases, Copenhagen, Denmark. Ticks Tick-Borne Dis.

[26] Kortela E, Kanerva M, Kurkela S, Oksi J, Järvinen A (2021). Suspicion of Lyme borreliosis in patients referred to an infectious diseases clinic: what did the patients really have?. Clin Microbiol Infect.

[27] Raffetin A, Schemoul J, Chahour A, Nguala S, Caraux-Paz P, Paoletti G (2022). Multidisciplinary management of suspected Lyme borreliosis: clinical features of 569 patients, and factors associated with recovery at 3 and 12 months, a prospective cohort study. Microorganisms.

[28] von Elm E, Altman D, Egger M, Pocock SJ, Gøtzsche PC, Vandenbroucke JP (2008). The strengthening the reporting of observational studies in epidemiology (STROBE) statement: guidelines for reporting observational studies. J Clin Epidemiol.

[29] Steere AC, Strle F, Wormser GP, Hu LT, Branda JA, Hovius JWR (2016). Lyme borreliosis. Nat Rev Dis Primer.

[30] De Paula DAG, Piatti NCTP, Costa LM, Chiavegato LD (2020). Satisfaction levels with physical therapy in hospitalized patients. Braz J Phys Ther.

[31] Bowling A, Rowe G, Lambert N, Waddington M, Mahtani KR, Kenten C (2012). The measurement of patients’ expectations for health care: a review and psychometric testing of a measure of patients’ expectations. Health Technol Assess Winch Engl.

[32] Wang X, Chen J, Burström B, Burström K (2019). Exploring pathways to outpatients’ satisfaction with health care in Chinese public hospitals in urban and rural areas using patient-reported experiences. Int J Equity Health.

[33] Kazemi A, Elfstrand Corlin T. Linking supportive leadership to satisfaction with care: proposing and testing a service-profit chain inspired model in the context of elderly care. J Health Organ Manag. 2021. 10.1108/JHOM-10-2020-0393. 10.1108/JHOM-10-2020-0393 33629577

[34] Moreno PI, Ramirez AG, San Miguel-Majors SL, Fox RS, Castillo L, Gallion KJ (2018). Satisfaction with cancer care, self-efficacy, and health-related quality of life in Latino cancer survivors. Cancer.

[35] Deguchi T, Takatsuna H, Yokoyama M, Shiosakai K, Inoue T, Seki H (2021). A cross-sectional web survey of satisfaction with treatment for pain in participants with suspected diabetic peripheral neuropathic pain in both feet. Adv Ther.

[36] Hofmann B (2002). On the triad disease, illness and sickness. J Med Philos.

[37] Haute Autorité de Santé (HAS) (2014). Parcours de soins et maladie chronique - Annonce et accompagnement du diagnostic d’un patient ayant une maladie chronique.

[38] Nakayama K, Osaka W, Matsubara N, Takeuchi T, Toyoda M, Ohtake N (2020). Shared decision making, physicians’ explanations, and treatment satisfaction: a cross-sectional survey of prostate cancer patients. BMC Med Inform Decis Mak.

[39] Elkbuli A, Stotsenburg M, Epstein C, Calvert K, Boneva D, McKenney M (2020). A multidisciplinary approach to improve pain management and satisfaction in a trauma population. J Trauma Nurs.

[40] O’Leary BD, Agnew GJ, Fitzpatrick M, Hanly AM (2019). Patient satisfaction with a multidisciplinary colorectal and urogynaecology service. Ir J Med Sci.

