# Supplementary material for: Acceptance of diagnosis and management satisfaction of patients with “suspected Lyme borreliosis” after 12 months in a multidisciplinary reference center: a prospective cohort study
Source: BMC Infect Dis. 2023 Jun 6;23:380. doi: 10.1186/s12879-023-08352-3 (PMC10243684; doi:10.1186/s12879-023-08352-3)
Supplement: Supplementary file 2 — Additional file 2. Satisfaction survey. [file 12879_2023_8352_MOESM2_ESM.docx]

**SATISFACTION SURVEY**

**Tick-Borne Diseases Reference Center of Paris and the Northern Region**

|  | Very dissatisfied / Dissatisfied / Neutral / Satisfied / Very satisfied  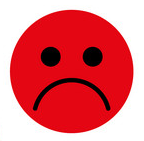 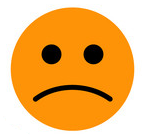 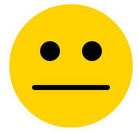 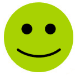 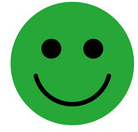 |
| --- | --- |
| **RECEPTION** | |
| By the secretary | 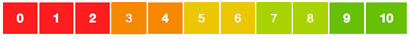 |
| **QUALITY OF CARE AND MANAGEMENT** | |
| By the medical team | 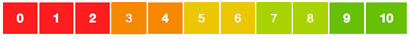 |
| By the paramedical team | 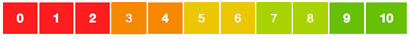 |
| Responsiveness and compassion to  patients | 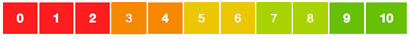 |
| Care path at TBD-RC | 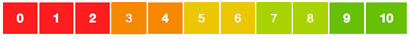 |
| **INFORMATION / EXPLANATIONS GIVEN TO THE PATIENTS** (have they answered your questions?) | |
| Given by the secretary | 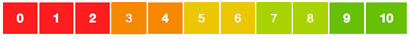 |
| Given by the paramedical team | 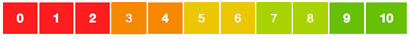 |
| Given by the medical team | 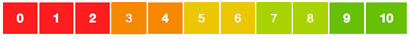 |
| **OVERALL APPRECIATION** | |
| Satisfaction of the final diagnosis | 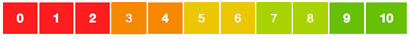 |
| Satisfaction of the global  management | 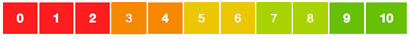 |
| Would you recommend the TBD-RC  to your surroundings? | 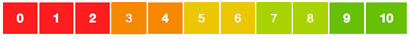 |
| What points did you enjoy? |  |
| What would you like us to change? |  |
| **CURRENT MEDICAL CONDITION** | |
| Acceptance of the final diagnosis | □ Yes □ Partially □ No |
| How would you assess your current  condition after the management at the  TBD-RC compared to the previous  one? | Very Bad / Worse / Unchanged / Good / Very good  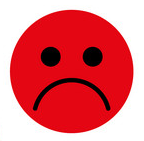 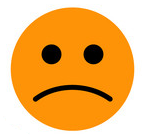 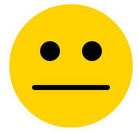 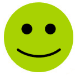 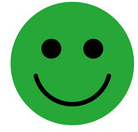  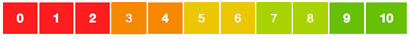 |
