# Supplementary material for: Acceptance of diagnosis and management satisfaction of patients with “suspected Lyme borreliosis” after 12 months in a multidisciplinary reference center: a prospective cohort study
Source: BMC Infect Dis. 2023 Jun 6;23:380. doi: 10.1186/s12879-023-08352-3 (PMC10243684; doi:10.1186/s12879-023-08352-3)
Supplement: Supplementary file 3 — Additional file 3: Supplementary file 2. Comparison of the epidemiological characteristics of the patients who answered or not to the satisfaction survey, consulting the TBD-RC of Paris and the Northern region. [file 12879_2023_8352_MOESM3_ESM.docx]

**Supplementary file 2.** Comparison of the epidemiological characteristics of the patients who answered or not to the satisfaction survey, consulting the TBD-RC of Paris and the Northern region.

| Epidemiological Characteristics of the Patients | Total  N = 569 (%) | Patients who answered to the satisfaction questionnaire  N=349 (%) | Patients who did not answer to the satisfaction questionnaire  N=220 (%) | *p*-Value |
| --- | --- | --- | --- | --- |
| Age, years (median [IQ 25,75]) | 48 [35,61] | 48 [35,62] | 48 [35.5,59] | 0.981 |
| Male | 220 (38.7) | 146 (41.8) | 74 (33.6) | 0.051 |
| Life style |  |  |  | 0.865 |
| Home in a rural area | 121 (21.2) | 72 (20.6) | 49 (22.3) | - |
| Employment in rural areas/forest | 30 (5.3) | 17 (4.9) | 13 (5.9) | - |
| Forest-based leisure activities | 399 (70) | 249 (71.4) | 150 (68.2) | - |
| No exposure | 19 (3.3) | 11 (3.2) | 8 (3.6) | - |
| Past history of tick-bite | 372 (65.3) | 234 (67.1) | 138 (62.7) | 0.291 |
| Past history of erythema migrans | 145 (25.4) | 97 (27.9) | 49 (22.3) | 0.137 |
| Patients referred by a physician  with a letter | 516 (90.7) |  |  | 0.726 |
| General Practitioner | 401 (70.4) | 241 (69.1) | 160 (72.7) | - |
| Specialist physician | 94 (16.5) | 59 (16.9) | 35 (15.9) | - |
| Emergency unit physician | 21 (3.7) | 13 (3.7) | 8 (3.6) | - |
| No letter, patient self-referral | 53 (9.5) | 36 (10.3) | 17 (7.7) | - |
| Duration (days) of chief complaints prior to examination at TBD-RC  (median [IQ 25,75]) | 512 [156,1392.5] | 425.5 [140.5, 1208.5] | 615 [168, 1535.5] | 0.064 |
| Patient’s chief complaint |  |  |  | 0.987 |
| Erythema migrans | 17 (3) | 10 (2.9) | 7 (3.2) |  |
| Clinical signs/symptoms implicating early disseminated LB (>six months) | 159 (27.9) | 100 (28.7) | 59 (26.8) |  |
| Clinical signs/symptoms implicating late disseminated LB (>six months) | 382 (67.2) | 232 (66.5) | 150 (68.2) |  |
| Questions after a tick-bite | 6 (1.1) | 4 (1.2) | 2 (0.9) |  |
| Positive serological test with no clinical signs | 5 (0.9) | 3 (0.9) | 2 (0.9) |  |
| Serological test |  |  |  | 0.913 |
| IgM and/or IgG positive in ELISA and WB | 180 (31.6) | 111 (31.8) | 69 (31.4) |  |
| Antibiotic therapy prescribed before TBD-RC | 369 (64.9) | 228 (65.3) | 141 (64.1) | 0.763 |
| Antibiotic therapy > 4 weeks | 117 (22.6) | 71 (20.3) | 46 (20.9) | 0.871 |
| Non-recommended treatments (>8 weeks of antibiotics and/or associated antimicrobials) | 101 (17.8) | 61 (17.5) | 40 (18.2) | 0.831 |
| Diagnosis retained at TBD-RC |  |  |  | 0.302 |
| Confirmed LB | 72 (12.7) | 48 (13.8) | 24 (10.9) | - |
| Possible LB | 43 (7.6) | 31 (8.9) | 12 (5.5) | - |
| PTLDS/sequelae | 58 (10.2) | 34 (9.7) | 24 (10.9) | - |
| Other diagnoses | 396 (69.6) | 236 (67.6) | 160 (72.7) | - |

LB = Lyme borreliosis; PTLDS = Post-Treatment Lyme Disease Syndrome; ELISA = Enzyme-Linked Immunosorbent Assay; WB = Western-Blot; TBD-RC = Tick-Borne Diseases Reference Center.
