# Supplementary material for: Acceptance of diagnosis and management satisfaction of patients with “suspected Lyme borreliosis” after 12 months in a multidisciplinary reference center: a prospective cohort study
Source: BMC Infect Dis. 2023 Jun 6;23:380. doi: 10.1186/s12879-023-08352-3 (PMC10243684; doi:10.1186/s12879-023-08352-3)
Supplement: Supplementary file 4 — Additional file 4. Multivariate analyses of the associated factors with the diagnostic acceptance versus no acceptance at 12 months. [file 12879_2023_8352_MOESM4_ESM.docx]

**Additional file 4. Multivariate analyses of the associated factors with the diagnostic acceptance versus no acceptance at 12 months**

| Risk Factor | n  (*N* = 349) | Diagnostic Acceptance  *n* (%) | Crude OR [95% CI] | *p*-Value | Adjusted OR [95% CI]* | *p*-Value |
| --- | --- | --- | --- | --- | --- | --- |
| Age (years) |  |  |  | 0.547 |  | 0.93 |
| <35 | 94 | 79 (84.0) | 1 |  | 1 |  |
| 35–48 | 82 | 62 (75.6) | 0.59 [0.28–1.25] |  | 0.74 [0.25–2.17] |  |
| 48–61 | 81 | 64 (79.0) | 0.71 [0.33–1.54] |  | 0.86 [0.28–2.59] |  |
| >61 | 92 | 75 (81.5) | 0.84 [0.39–1.80] |  | 0.74 [0.24–2.24] |  |
| Sex |  |  |  | 0.971 |  | 0.91 |
| Male | 146 | 117 (80.1) | 0.99 [0.58–1.69] |  | 1.05 [0.47–2.36] |  |
| Female | 203 | 163 (80.3) | 1 |  | 1 |  |
| History of tick-bite |  |  |  | 0.719 | - | - |
| Yes | 234 | 189 (80.8) | 1.11 [0.64–1.93] |  |  |  |
| No | 115 | 91 (79.1) | 1 |  |  |  |
| History of erythema migrans |  |  |  | 0.225 | - | - |
| Yes | 97 | 82 (84.5) | 1.46 [0.78–2.74] |  |  |  |
| No | 251 | 198 (78.9) | 1 |  |  |  |
| Missing data | 1 | 1 (100.0) | - |  |  |  |
| Serology |  |  |  | 0.930 | - | - |
| Positive serology in ELISA and WB | 111 | 45 (29.2) | 1.17 [0.63–2.16] |  |  |  |
| Positive serology in ELISA only | 46 | 19 (29.2) | 1.06 [0.47–2.40] |  |  |  |
| Negative serology in ELISA | 166 | 132 (79.5) | 1 |  |  |  |
| Patient with no serology (erythema migrans) | 26 | 15 (53.6) | 0.86 [0.32–2.30] |  |  |  |
| Delay 1st symptoms-1st consultation at the TBD-RC |  |  |  | 0.024 | - | - |
| 0–155 days (0.0–0.4 year) | 93 | 84 (90.3) | 1 |  |  |  |
| 155–512 days (0.4–1.4 years) | 96 | 72 (75.0) | 0.32 [0.14–0.74] |  |  |  |
| 512–1393 days (1.4–3.8 years) | 81 | 63 (77.8) | 0.38 [0.16–0.89] |  |  |  |
| >1393 days (>3.8 years) | 78 | 60 (76.9) | 0.36 [0.15–0.85] |  |  |  |
| Missing data | 1 | 1 (100.0) | - |  |  |  |
| Delay 1st consultation at the TBD-RC-final diagnosis |  |  |  | 0.155 | - | - |
| 0 day | 142 | 119 (83.8) | 1 |  |  |  |
| 1–15 days | 23 | 20 (87.0) | 1.29 [0.35–4.69] |  |  |  |
| 15–83 days | 95 | 69 (72.6) | 0.51 [0.27–0.97] |  |  |  |
| >83 days | 88 | 72 (81.8) | 0.87 [0.43–1.75] |  |  |  |
| Missing data | 1 | 1 (100.0) | - |  |  |  |
| Final diagnosis |  |  |  | <0.001 |  | 0.031 |
| Confirmed LB | 48 | 47 (97.9) | 13.00 [1.75–96.2] |  | 4.17 [0.51-34.07] |  |
| Possible LB | 31 | 24 (77.4) | 0.95 [0.39–2.32] |  | 0.23 [0.07-0.77] |  |
| PTLDS or sequelae | 34 | 24 (70.6) | 0.66 [0.30–1.47] |  | 0.55 [0.16-1.89] |  |
| Other diagnoses | 236 | 185 (78.4) | 1 |  | 1 |  |
| Number of diagnosis per patient |  |  |  | 0.014 | - | - |
| 1 diagnosis | 191 | 164 (85.9) | 1 |  |  |  |
| 2 diagnoses | 98 | 71 (72.5) | 0.43 [0.24–0.79] |  |  |  |
| ≥3 diagnoses | 60 | 45 (75.0) | 0.49 [0.24–1.01] |  |  |  |
| Antibiotics prescribed before the TBD-RC |  |  |  | 0.263 | - | - |
| Yes | 228 | 179 (78.5) | 0.72 [0.41–1.28] |  |  |  |
| No | 121 | 101 (83.5) | 1 |  |  |  |
| History of non-recommended antibiotics |  |  |  | 0.043 | - | - |
| Yes | 61 | 43 (70.5) | 0.51 [0.27–0.96] |  |  |  |
| No | 288 | 237 (82.3) | 1 |  |  |  |
| First line of antibiotics prescribed at the TBD-RC |  |  |  | 0.025 | - | - |
| Yes | 61 | 43 (70.5) | 2.04 [1.06–3.92] |  |  |  |
| No | 288 | 237 (82.3) | 1 |  |  |  |
| Second line of antibiotics at the TBD-RC |  |  |  | 0.765 | - | - |
| Yes | 13 | 10 (76.9) | 0.81 [0.22–3.04] |  |  |  |
| No | 336 | 270 (80.4) | 1 |  |  |  |
| Care and quality of management by the medical team |  |  |  | <0.001 |  | 0.008 |
| Not satisfied (score 0-4) | 9 | 1 (11.1] | 0.01 [0.01-0.29] |  | 0.09 [0.01-1.61] |  |
| Moderately satisfied (score 5-6) | 22 | 2 (9.1) | 0.03 [0.01-0.12] |  | 0.05 [0.01-0.32] |  |
| Satisfied (score 7-8) | 85 | 67 (78.8) | 1 |  | 1 |  |
| Very satisfied (score 9-10) | 231 | 210 (90.9) | 2.69 [1.35-5.34] |  | 0.72 [0.25-2.04] |  |
| Missing data | 2 | 0 (0.0) | - |  | - |  |
| Responsiveness and compassion to patients |  |  |  | <0.001 | - | NI |
| Not satisfied (score 0-4) | 9 | 2 (22.2) | 0.09 [0.02-0.49] |  |  |  |
| Moderately satisfied (score 5-6) | 23 | 4 (17.4) | 0.07 [0.02-0.23] |  |  |  |
| Satisfied (score 7-8) | 89 | 67 (75.3) | 1 |  |  |  |
| Very satisfied (score 9-10) | 225 | 206 (91.6) | 3.56 [1.82-6.98] |  |  |  |
| Missing data | 3 | 1 (33.3) | - |  |  |  |
| Care-path at TBD-RC |  |  |  | <0.001 |  | 0.041 |
| Not satisfied (score 0-4) | 11 | 2 (18.2) | 0.08 [0.02-0.38] |  | 1.00 [0.08-13.15] |  |
| Moderately satisfied (score 5-6) | 25 | 4 (16.0) | 0.07 [0.02-0.21] |  | 0.54 [0.11-2.53] |  |
| Satisfied (score 7-8) | 101 | 75 (74.3) | 1 |  | 1 |  |
| Very satisfied (score 9-10) | 208 | 196 (94.2) | 5.66 [2.72- 11.80] |  | 4.64 [1.52-14.16] |  |
| Missing data | 4 | 3 (0.8) | - |  | - |  |
| Information and explanations given to the patients by the medical team |  |  |  | <0.001 | - | NI |
| Not satisfied (score 0-4) | 11 | 1 (9.1) | 0.04 [0.01-0.34] |  |  |  |
| Moderately satisfied (score 5-6) | 24 | 6 (25.0) | 0.14 [0.05-0.39] |  |  |  |
| Satisfied (score 7-8) | 82 | 58 (70.7) | 1 |  |  |  |
| Very satisfied (score 9-10) | 230 | 214 (93.0) | 5.53 [2.76- 11.10] |  |  |  |
| Missing data | 2 | 1 (50.0) | - |  |  |  |
| Current medical condition assessed by the patient compared to before the TBD-RC |  |  |  | <0.001 |  | <0.001 |
| Deterioration (score 0-4) | 10 | 4 (40.0) | 0.12 [0.03-0.45] |  | 0.18 [0.03-1.03] |  |
| Stagnation (score 5-6) | 54 | 19 (35.2) | 0.10 [0.05-0.20] |  | 0.16 [0.06-0.42] |  |
| Partial improvement (score 7-8) | 140 | 119 (85.0) | 1 |  | 1 |  |
| Recovery (score 9-10) | 145 | 138 (95.2) | 3.48 [1.43- 8.47] |  | 2.66 [0.90-7.88] |  |

TBD-RC = Tick-Borne Diseases Reference Center; PTLDS = Post-Treatment Lyme Disease Syndrome; NI = not included to avoid collinearity in the model; *Factors associated with the outcome with a *p*-value <0.25 in univariate analysis were considered in the multivariate model. Age and gender were forced in the model.
