# Supplementary material for: Acceptance of diagnosis and management satisfaction of patients with “suspected Lyme borreliosis” after 12 months in a multidisciplinary reference center: a prospective cohort study
Source: BMC Infect Dis. 2023 Jun 6;23:380. doi: 10.1186/s12879-023-08352-3 (PMC10243684; doi:10.1186/s12879-023-08352-3)
Supplement: Supplementary file 5 — Additional file 5. Multivariate analyses of the associated factors with the management satisfaction versus no satisfaction at 12 months. [file 12879_2023_8352_MOESM5_ESM.docx]

**Additional file 5. Multivariate analyses of the associated factors with the management satisfaction versus no satisfaction at 12 months.**

| Risk Factor | n  (*N* = 349) | Satisfaction of the management *n* (%) | Crude OR [95% CI] | *p*-Value | Adjusted OR [95% CI]* | *p*-Value |
| --- | --- | --- | --- | --- | --- | --- |
| Age (years) |  |  |  | 0.346 |  | 0.051 |
| <35 | 94 | 78 (83.0) | 1 |  | 1 |  |
| 35–48 | 82 | 66 (80.5) | 0.85 [0.39–1.82] |  | 2.40 [0.61–9.48] |  |
| 48–61 | 81 | 71 (87.7) | 1.46 [0.62–3.42] |  | 31.98 [1.79–571.74] |  |
| >61 | 92 | 82 (89.1) | 1.68 [0.72–3.93] |  | 85.74 [1.53–4815.75] |  |
| Sex |  |  |  | 0.495 |  | 0.839 |
| Male | 146 | 122 (83.6) | 0.81 [0.45–1.47] |  | 0.87 [0.23–3.25] |  |
| Female | 203 | 175 (86.2) | 1 |  | 1 |  |
| Delay 1st symptoms-1st consultation at the TBD-RC |  |  |  | 0.155 |  | - |
| 0–155 days (0.0–0.4 year) | 93 | 85 (85.2) | 1 |  | - |  |
| 155–512 days (0.4–1.4 years) | 96 | 81 (84.4) | 0.51 [0.20–1.26] |  | - |  |
| 512–1393 days (1.4–3.8 years) | 81 | 68 (84.0) | 0.49 [0.19–1.26] |  | - |  |
| >1393 days (>3.8 years) | 78 | 62 (79.5) | 0.36 [0.15–0.91] |  | - |  |
| Missing data | 1 | 1 (100.0) | - |  | - |  |
| Delay 1st consultation at the TBD-RC-final diagnosis |  |  |  | 0.332 |  | - |
| 0 day | 142 | 121 (83.8) | 1 |  | - |  |
| 1–15 days | 23 | 22 (95.7) | 3.82 [0.49–29.86] |  | - |  |
| 15–83 days | 95 | 78 (82.1) | 0.80 [0.40–1.60] |  | - |  |
| >83 days | 88 | 76 (86.4) | 1.10 [0.51-2.36] |  | - |  |
| Missing data | 1 | 1 (100.0) | - |  | - |  |
| Initial orientation |  |  |  | 0.066 |  | - |
| Outpatient management | 186 | 158 (85.0) | 1 |  | - |  |
| One-day-hospitalization | 95 | 76 (80.0) | 0.71 [0.37-1.35] |  | - |  |
| Hospitalization | 68 | 63 (92.7) | 2.23 [0.83-6.04] |  | - |  |
| Final diagnosis |  |  |  | 0.003 |  | - |
| Confirmed LB | 48 | 47 (97.9) | 10.18 [1.37-75.83] |  | - |  |
| Possible LB | 31 | 29 (93.6) | 3.14 [0.72-13.67] |  | - |  |
| PTLDS or sequelae | 34 | 27 (79.4) | 0.84 [0.34-2.05] |  | - |  |
| Other diagnoses | 236 | 194 (82.2) | 1 |  | - |  |
| First line of antibiotics prescribed at the TBD-RC |  |  |  | 0.011 |  | - |
| Yes | 103 | 95 (92.2) | 2.59 [1.17–5.71] |  | - |  |
| No | 246 | 202 (82.1) | 1 |  | - |  |
| Satisfaction of the reception by the secretary |  |  |  | <0.001 |  | NI |
| Not satisfied (score 0-4) | 6 | 0 (0.0) | 0.11 [0.05-0.27] |  | - |  |
| Moderately satisfied (score 5-6) | 24 | 12 (50.0) |  |  | - |  |
| Satisfied (score 7-8) | 130 | 111 (85.4) | 1 |  | - |  |
| Very satisfied (score 9-10) | 178 | 164 (92.1) | 2.01 [0.97-4.17] |  | - |  |
| Missing data | 11 | 10 (90.9) | - |  | - |  |
| Care and quality of management by the paramedical team |  |  |  | <0.001 |  | NI |
| Not satisfied (score 0-4) | 0 | 0 (0.0) | 0.02 [0.01-0.16] |  | - |  |
| Moderately satisfied (score 5-6) | 14 | 1 (7.1) |  |  | - |  |
| Satisfied (score 7-8) | 62 | 50 (80.7) | 1 |  | - |  |
| Very satisfied (score 9-10) | 128 | 122 (95.3) | 4.88 [1.74-13.72] |  | - |  |
| Missing data | 145 | 124 (85.5) | - |  | - |  |
| Care and quality of management by the medical team |  |  |  | <0.001 |  | 0.001 |
| Not satisfied (score 0-4) | 9 | 0 (0.0] | 0.01 [0.00-0.04] |  | 0.01 [0.00-0.10] |  |
| Moderately satisfied (score 5-6) | 22 | 1 (4.6) |  |  |  |  |
| Satisfied (score 7-8) | 85 | 73 (85.9) | 1 |  | 1 |  |
| Very satisfied (score 9-10) | 231 | 222 (96.1) | 4.05 [1.64-10.01] |  | 1.30 [0.22-7.66] |  |
| Missing data | 2 | 1 (50.0) | - |  | - |  |
| Responsiveness and compassion to patients |  |  |  | <0.001 |  | - |
| Not satisfied (score 0-4) | 9 | 1 (11.1) | 0.01 [0.00-0.04] |  | - |  |
| Moderately satisfied (score 5-6) | 23 | 0 (0.0) |  |  | - |  |
| Satisfied (score 7-8) | 89 | 76 (85.4) | 1 |  | - |  |
| Very satisfied (score 9-10) | 225 | 218 (96.9) | 5.33 [2.05-13.85] |  | - |  |
| Missing data | 3 | 2 (66.7) | - |  | - |  |
| Care-path at TBD-RC |  |  |  | <0.001 |  | 0.009 |
| Not satisfied (score 0-4) | 11 | 1 (9.1) | 0.01 [0.00-0.11] |  | 1.13 [0.03-43.02] |  |
| Moderately satisfied (score 5-6) | 25 | 2 (8.0) | 0.01 [0.00-0.06] |  | 0.01 [0.00-0.08] |  |
| Satisfied (score 7-8) | 101 | 89 (88.1) | 1 |  | 1 |  |
| Very satisfied (score 9-10) | 208 | 202 (97.1) | 4.54 [1.65-12.48] |  | 0.33 [0.05-2.10] |  |
| Missing data | 4 | 3 (75.0) | - |  | - |  |
| Information and explanations given to the patients by the secretary |  |  |  | <0.001 |  | NI |
| Not satisfied (score 0-4) | 9 | 1 (11.1) | 0.03 [0.01-0.23] |  | - |  |
| Moderately satisfied (score 5-6) | 21 | 7 (33.3) | 0.11 [0.04-0.31] |  | - |  |
| Satisfied (score 7-8) | 110 | 90 (81.8) | 1 |  | - |  |
| Very satisfied (score 9-10) | 160 | 155 (96.9) | 6.89 [2.50-18.99] |  | - |  |
| Missing data | 49 | 44 (89.8) | - |  | - |  |
| Information and explanations given to the patients by the paramedical team |  |  |  | <0.001 |  | NI |
| Not satisfied (score 0-4) | 0 | 0 (0.0) | 0.05 [0.01-0.18] |  | - |  |
| Moderately satisfied (score 5-6) | 20 | 3 (15.0) |  |  | - |  |
| Satisfied (score 7-8) | 63 | 50 (79.4) | 1 |  | - |  |
| Very satisfied (score 9-10) | 124 | 121 (86.6) | 10.49 [2.86-38.40] |  | - |  |
| Missing data | 142 | 123 (86.6) | - |  | - |  |
| Information and explanations given to the patients by the medical team |  |  |  | <0.001 |  | 0.001 |
| Not satisfied (score 0-4) | 11 | 0 (0.0) | 0.01 [0.00-0.06] |  | 0.02 [0.00-0.49] |  |
| Moderately satisfied (score 5-6) | 24 | 2 (8.3) |  |  |  |  |
| Satisfied (score 7-8) | 82 | 68 (82.9) | 1 |  | 1 |  |
| Very satisfied (score 9-10) | 230 | 225 (97.8) | 9.26 [3.22-26.65] |  | 23.39 [3.52-155.54] |  |
| Missing data | 2 | 2 (100.0) | - |  | - |  |

TBD-RC = Tick-Borne Diseases Reference Center; PTLDS = Post-Treatment Lyme Disease Syndrome; NI = Not included to avoid collinearity in the regression model. ; *Factors associated with the outcome with a *p*-value <0.25 in univariate analysis were considered in the multivariate model. We focused on the medical management only, which seemed more relevant in this study, especially as we then studied the concordance of the health status assessed by doctors and patients. Age and gender were forced in the model.
